# Targeting High Dynamin-2 (*DNM2*) Expression by Restoring Ikaros Function in Acute Lymphoblastic Leukemia

**DOI:** 10.1038/srep38004

**Published:** 2016-11-25

**Authors:** Zheng Ge, Yan Gu, Qi Han, Gang Zhao, Min Li, Jianyong Li, Baoan Chen, Tianyu Sun, Sinisa Dovat, Robert Peter Gale, Chunhua Song

**Affiliations:** 1 Department of Hematology, Zhongda Hospital, Medical School of Southeast University, Nanjing 210009, China; 2 Department of Hematology, The First Affiliated Hospital of Nanjing Medical University, Jiangsu Province Hospital, Nanjing 210029, China; 3 Lewis Katz School of Medicine, Temple University, Philadelphia, PA 19140, USA; 4 Department of Pediatrics, Pennsylvania State University Medical College, Hershey, PA 17033, USA; 5 Haematology Research Center, Division of Experimental Medicine, Department of Medicine, Imperial College London, London, SW7 2AZ, UK

## Abstract

Dynamin-2 (*DNM2*) is a GTPase essential for intracellular vesicle formation and trafficking, cytokinesis and receptor endocytosis. Mutations in *DNM2* are common in early T-cell precursor acute lymphoblastic leukemia. However, *DNM2* expression in other types of ALL are not reported. We studied *DNM2* mRNA level in adults with B- and T-cell ALL. We found *DNM2* is more highly expressed compared with normals in both forms of ALL. High *DNM2* expression is associated with some clinical and laboratory features, inferior outcomes and with leukaemia cell proliferation. We also found Ikaros directly binds the *DNM2* promoter and suppresses *DNM2* expression. Consequently *IKZF1* deletion is associated with high *DNM2* expression. Conversely, casein kinase-2 (CK2)-inhibitor increases Ikaros function thereby inhibiting *DNM2* expression. Inhibiting *DNM2* suppresses proliferation of leukemia cells and synergizes with CK2 inhibition. Our data indicate high *DNM2* expression is associated with Ikaros dysregulation and may be important in the development of B-ALL.

Dynamin-2 (*DNM2*) is a microtubule-associated GTPase essential for intracellular vesicle formation and trafficking, cytokinesis, and receptor endocytosis. Recent reports suggest over-expression of *DNM2* promotes cancer cell growth, migration and invasion in diverse cancers^1^,^2^,^3^. One DNM2 mutant, *DNM*2^V265G^, is associated with cancer development in mice^4^. Recently, mutations in *DNM2* were detected in early T-cell precursor acute lymphoblastic leukemia (ALL)^5^,^6^. There are no studies of *DNM2* mRNA levels in ALL.

*IZKFI* encodes the DNA-binding zinc finger protein Ikaros essential for normal hematopoiesis and immune development^7^,^8^,^9^,^10^,^11^. Ikaros is also a tumor suppressor gene in acute B- and T-cell ALL^11^,^12^,^13^,^14^. Recently, we reported the Ikaros global binding profiling in ALL cells. We found Ikaros regulates expression of its targets through chromatin remodeling in ALL^15^,^16^,^17^,^18^. We also found CK2-inhibitors increase tumor suppressor activity of Ikaros and act as a functional activator of Ikaros^15^,^16^,^17^. Our ChIP-seq data indicate Ikaros binding peaks in the promoter region of *DNM2*. However, it is unclear how Ikaros regulates *DNM2* expression. We studied correlations between *DNM2* mRNA level and outcomes in adults with B- and T-cell ALL. Our data suggest high expression of *DNM2* with consequent Ikaros dysfunction is associated with development of B-cell ALL.

## Results

### Clinical and laboratory variables in subjects with high and low *DNM2* expression

**Figure 1:**
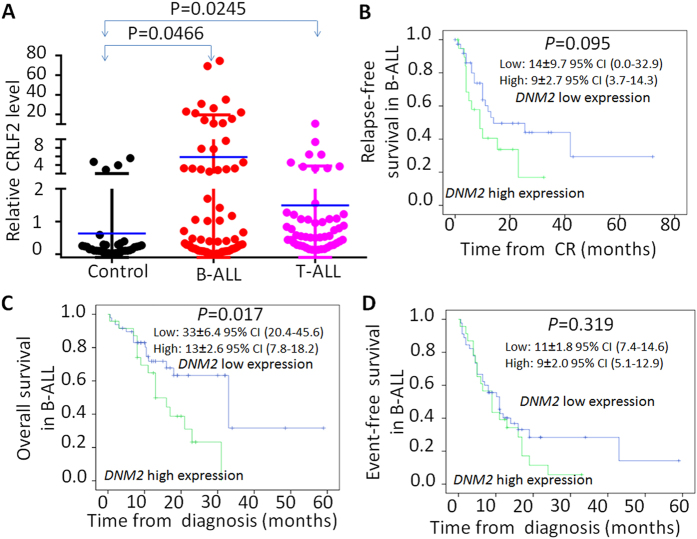
*DNM2* expression in ALL patients and its correlation with survival of B-ALL patients. q-PCR was performed to detect *DNM2* in ALL patient samples and normal BM controls. (**A**) Comparison of *DNM2* expression in B-ALL and T-ALL to normal BM control; (**B–D**) Comparison of relapse-free survival (**B**), overall survival (**C**) and event-free survival in patients with *DNM2* high expression to those in patients with *DNM2* low expression.

*DNM2* mRNA levels in bone marrow samples from adults with ALL, especially those with B-cell ALL, were significantly higher than those in normals (Fig. 1A). We compared clinical and laboratory variables in subjects with B- and T-cell ALL divided into cohorts with high or low *DNM2* mRNA levels (Tables 1 and 2). In B-cell ALL, high *DNM2* mRNA levels were associated with a WBC ≥ 30 × 10E + 9/L compared to low *DNM2* expression (79% *vs.* 42%; *P* = 0.003). This associated was confirmed in multivariate analyses (HR 4.56, 95% confidence interval [CI] 1.09, 19.01; *P* = 0.037; Table 1). The high *DNM2* expression cohort also had a higher frequency of lymph-adenopathy compared with the low *DNM2* expression cohort (61% *vs.* 23%; *P* = 0.002) confirmed in multivariate analyses (HR 7.245, [1.74, 30.13]; *P* = 0.006; Table 1). There were no significant associations between clinical and laboratory variables in the high *versus* low *DNM2* expression cohorts in subjects with T-cell ALL (Table 2).

### Correlation between *DNM2* expression and clinical outcomes

Subjects with B-cell ALL and high *DNM2* expression had briefer median RFS than those with low *DNM2* expression (9 months [3.7, 14.3 months] *vs.* 14 months [0, 37.9 months]; *P* = 0.095; Fig. 1B). 5 year survival was significantly briefer in those with high *DNM2* expression (13 months [7.8, 18.2 months] *vs.* 33 months [20.4, 45.6 months]; *P* = 0.017; Fig. 1C). There was no significant difference in median EFS between the cohorts (9 months [5.1, 12.9 months] *vs.* 11 months [7.4, 14.6 months]; *P* = 0.319; Fig. 1D). There were no significant associations between *DNM2* expression and any outcome in subjects with T-ALL (Supplemental Figure 2 and Table 2).

### Ikaros binds to the *DNM2* promoter and regulates its expression in ALL

**Figure 2:**
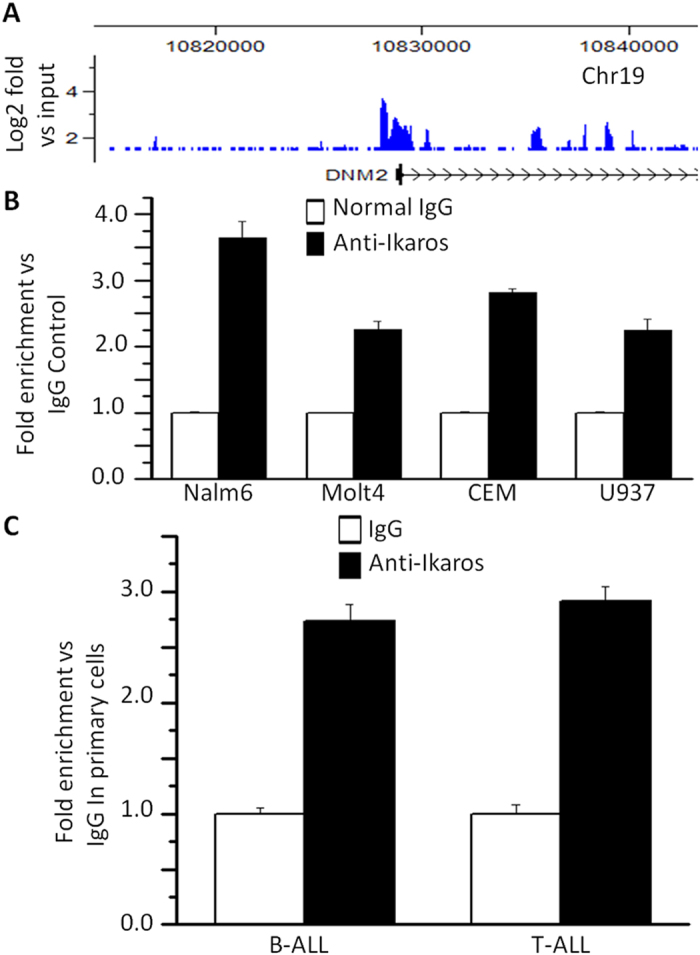
Ikaros binds the promoters of *DNM2*. (**A**) *Ikaros* binding peaks at the promoter of DNM2 identified by ChIP-seq in Nalm6 cells. (**B,C**) qChIP assay to assess Ikaros binding at the promoter of *DNM2* in ALL cell lines (**B**) and primary ALL patients’ samples(**C**).

**Figure 3:**
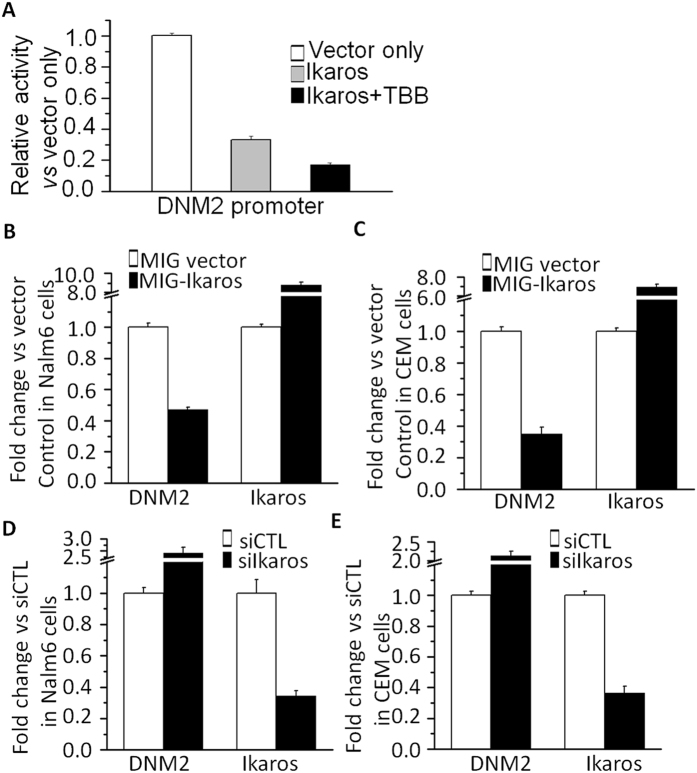
Ikaros suppresses *DNM2* expression. (**A**) The promoter activity of *DNM2* promoters by luciferase reporter assay following transfection with *Ikaros* or control vector in HEK293 cells. (**B,C**) Expression of *DNM2* in Nalm6 (**B**) and CEM (**C**) cells transduced with vector containing Ikaros as compared to control vector. (**D,E**) Comparison of *DNM2* expression in Nalm6 (**D**) and CEM (**E**) cells treated with Ikaros shRNA (siIkaros) or scramble shRNA (siCTL). Gene expression is determined by RT-qPCR using total RNA isolated from the cells transfected with scramble shRNA (siCTL) or *Ikaros* shRNA (siIkaros) for 2 days. Compared with siCTL in C: *p < 0.05, **p < 0.01 compared to siCTL group.

**Figure 4:**
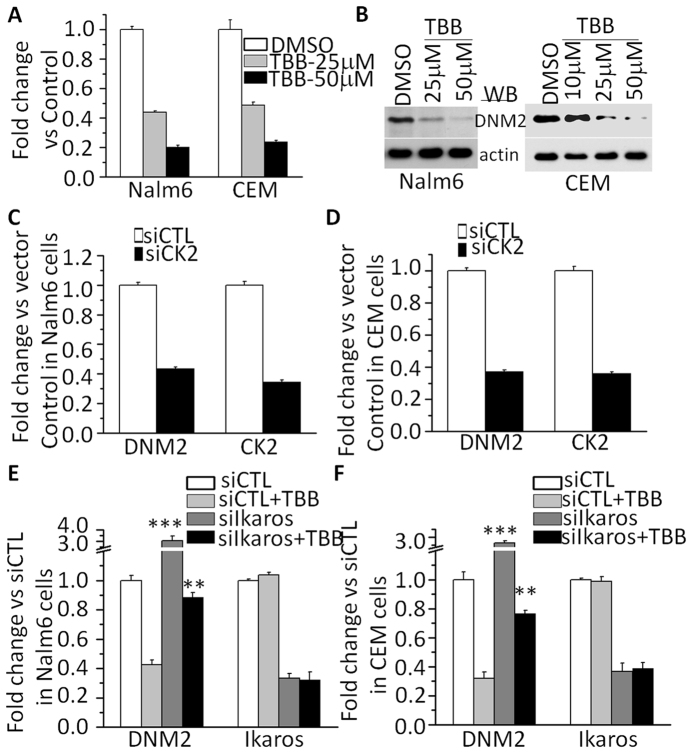
Effect of CK2 inhibitor on expression of *DNM2*. (**A,B**) The CK2 inhibitor, TBB, suppresses *DNM2* expression in ALL cells as assessed by q-PCR (**A**) and by western blot (**B**). (**C,D**) CK2 knockdown^15^ suppresses *DNM2* expression in Nalm6 B-ALL (**C**) and CEM T-ALL (**D**) cells by q-PCR. (**E,F**) Ikaros knockdown rescues the TBB-induced change in *DNM2* in Nalm6 B-ALL (**E**) and CEM T-ALL (**F**) cells. Compared with siCTL + TBB in D and E: *P < 0.05; **P < 0.01.

To address the potential mechanism underlying high *DNM2* expression we analyzed transcription factor motifs in the *DNM2* promoter region. ChIP-seq data identified Ikaros binding peaks in the *DNM2* promoter region in Nalm6 B-ALL (Fig. 2A) and primary B-cell ALL cells (Supplemental Figure 3)^15^,^16^. Ikaros binding was confirmed by qChIP assay (Fig. 2B and C). Weak binding was also found in U-937 AML cells and Molt-4 T-cell ALL cells (Fig. 2B). Ikaros suppressed promoter activity of *DNM2* by luciferase reporter assay (Fig. 3A). These data indicate a direct effect of Ikaros on *DNM2* transcription. Expression of Ikaros suppresses *DNM2* mRNA levels in Nalm6 (Fig. 3B) and CEM cells (Fig. 3C). Conversely, efficient Ikaros knockdown increased *DNM2* expression in Nalm6 (Fig. 3D) and CEM cells (Fig. 3E). Treating Nalm6 and CEM cells with TBB suppressed *DNM2* mRNA levels in a dose-dependent manner detected by qPCR (Fig. 4A) and protein levels by western blotting (Fig. 4B). CK2 knockdown with shRNA also induced suppression of *DNM2* expression in Nalm6 (Fig. 4C) and CEM cells (Fig. 4D). Ikaros knockdown with shRNA blocked the TBB-induced decrease of *DNM2* expression in Nalm6 and CEM (Fig. 4E and F) cells. These data indicate *DNM2* is the direct target of Ikaros and that Ikaros suppresses *DNM2* expression in B- and T-cell ALL.

### Correlation of *IKZF1* deletion with *DNM2* high expression in ALL cells

**Figure 5:**
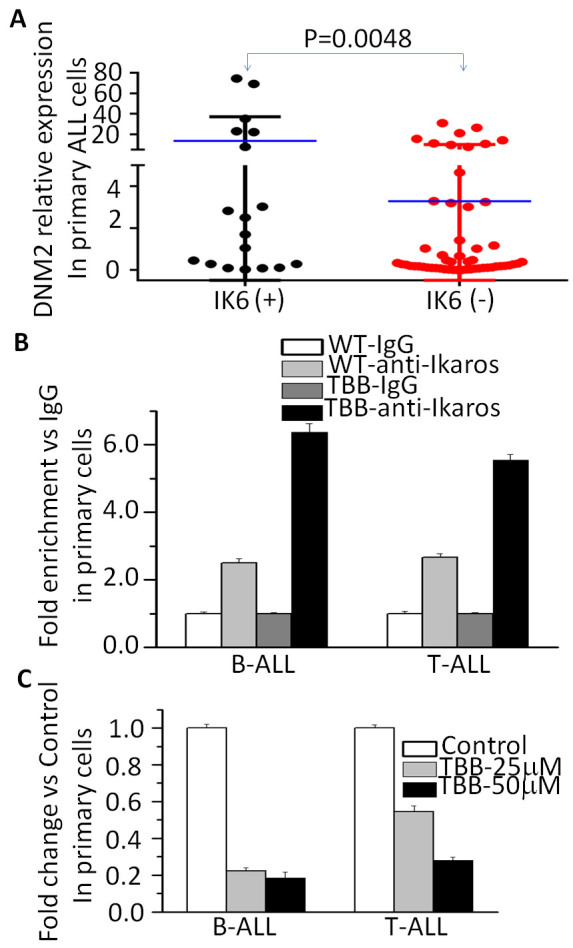
*IKZF1* deletion results in increase of *DNM2* expression in primary B-ALL cells. (**A**) comparison of *DNM2 expression* in patients with or without *IKZF1* deletion; (**B**) Effect of CK2 inhibitor (TBB) on Ikaros binding on the promoter of DNM2 in primary ALL cells; (**C**) Effect of CK2 inhibitor (TBB) on expression of *DNM2* in primary B-ALL cells with TBB treatment for 2 days.

We next analyzed a possible correlation between *IKZF1* levels with *DNM2* in primary ALL cells^19^,^20^,^21^. We found *IKZF1* mRNA levels were inversely-correlated with high *DNM2* expression in both cohorts (Supplemental Figures 4 and 5). We also found *DNM2* expression was significantly higher in B-cell ALL cells with Ik6 (13.58 ± 5.53 *vs.* 3.28 ± 0.92; P = 0.0048; Fig. 5A). Frequency of Ik6 in subjects with high *DNM2* expression was significantly higher than in subjects with low *DNM2* expression (54% *vs.* 21%; *P* = 0.004) confirmed in multivariate analyses (HR 6.15 [1.40, 27.00]; *P* = 0.016; Table 1). These data suggest *IKZF1* deletion may contribute to high *DNM2* expression. Furthermore, treatment of primary B- and T-cell ALL cells with TBB increased Ikaros binding to the *DNM2* promoter region compared with normals (Fig. 5B). qPCR assays showed TBB treatment inhibited expression of *DNM2* mRNA in a dose-dependent manner (Fig. 5C). These results indicate Ikaros binds to the *DNM2* promoter and treatment with TBB, which enhances Ikaros tumor suppressor activity, suppresses *DNM2* expression.

### Enhanced Ikaros activity from CK2-inhibition increases H3K9me3 at the *DNM2* promoter

**Figure 6:**
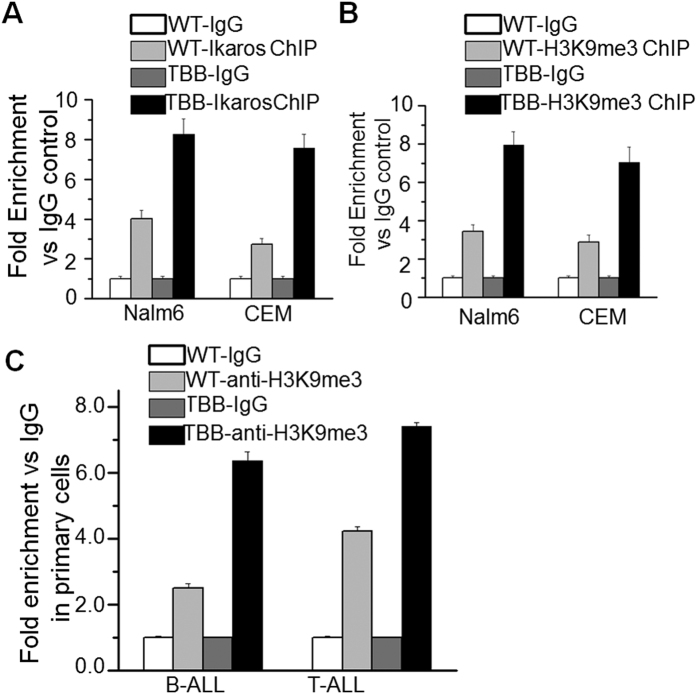
Ikaros suppresses *DNM2* expression via chromatin remodeling. (**A–C**) The CK2 inhibitor, TBB, increases the enrichment of Ikaros (**A**) and H3K9me3 (**B,C**) in B-ALL cells and patients’ samples. The cells were treated with TBB 25 μM for 1–2 days and qChIP was performed as described in methods section.

We reported Ikaros regulates gene expression through chromatin remodeling^16^. To further explore the epigenetic mechanism by which Ikaros regulates *DNM2* expression we performed ChIP assay and amplified the resulting *DNM2* promoter sequences. Our data indicate TBB treatment significantly increases binding of Ikaros to the *DNM2* promoter region compared with untreated Nalm6 B-cell ALL cells and with CEM T-cell ALL cells (Fig. 6A). TBB treatment also enhanced binding of H3K9me^3^ at the *DNM2* promoter region in Nalm6 and CEM cells (Fig. 6B). Results were similar with primary B- and T-cell ALL cells (Fig. 6C). There was no enrichment of other histone modification markers in the *DNM2* promoter region (data not shown).

### MiTMAB suppresses cell proliferation and synergizes with CK2-inhibitors in ALL

**Figure 7:**
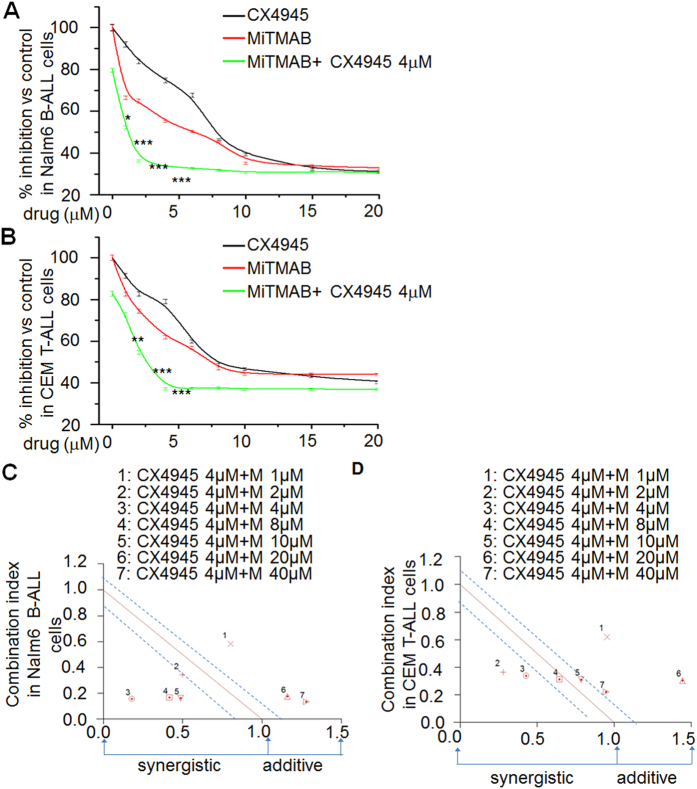
DNM2 inhibitor suppresses the cell proliferation of ALL cells and its synergistic effect with CK2 inhibitors. (**A,B**) Effect of CX-4945 (grey line), MiTMAB (red line) and combination of MiTMAB with CX-4945 (green line) on proliferation of Nalm6 cells (**A**) and CEM cells (**B**). *P ≤ 0.05; **P ≤ 0.01; *** ≤ 0.001. (**C,D**) Analysis of synergistic effect of MiTMAB with CX4945 with Calcusyn in Nalm6 cell (**C**) and CEM cells (**D**). Y axis is the combination index (CI) value. CI value is: 1.15 to 0.8, additive effect; 0.85 to 0.7, moderate synergistic; < 0.7, very synergistic effect.

We used the WST-1 cell proliferation assay to test whether targeting *DNM2*, with MiTMAB, a DNM2-inhibitor, had an anti-leukemia effect in ALL cells^22^. Treatment with MiTMAB had a dose-dependent effect on proliferation of Nalm6 B-cell ALL cells (Fig. 7A) and CEM T-cell ALL cells (Fig. 7B). In contrast, treatment with Dynasore, a GTPase-inhibitor which suppresses dynamin activity and prevents endocytosis, had no significant effect on cell proliferation (data not shown)^23^,^24^. Because CK2-inhibition results in reduced expression of *DNM2* we tested whether the CK2-inhibitors TBB and CX-4945 with MiTMAB altered cell proliferation. CX-4945 (4 μM) and various doses of MiTMAB resulted in less proliferation of Nalm-6 (Fig. 7A) and CEM cells (Fig. 7B) compared to MiTMAB alone (Fig. 7A green line *vs*. red line). Using the CalcuSyn assay we found treatment with CX-4945 and MiTMAB were synergistic in inhibiting proliferation of Nalm-6 (Fig. 7C) and CEM (Fig. 7D) cells^25^. Similar result was observed for TBB (data not shown). These data indicate targeting *DNM2* transcription significantly suppresses proliferation of ALL cells and that inhibiting *DNM2* with TBB, a CK2–inhibitor restores Ikaros function.

## Discussion

**Figure 8:**
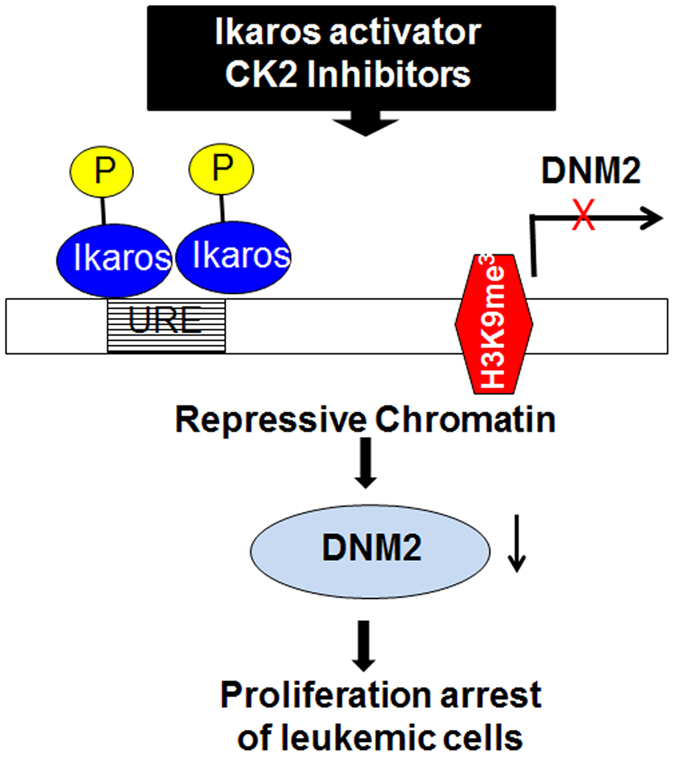
Model for the mechanism of Ikaros regulation of *DNM2* expression.

High *DNM2* expression was seen in adults with B- and T-cell ALL. In persons with B-cell ALL, high *DNM2* expression is associated with cell proliferation, extra-medullary leukaemia, an increased relapse risk and briefer survival. We also found *DNM2* expression is significantly higher in subjects with *IKZF1* deletion. Ikaros directly suppresses *DNM2* expression in ALL cells and a CK2-inhibitor which restores Ikaros function and suppresses *DNM2* expression in an Ikaros-dependent manner in leukemia cells by recruiting the repressive histone marker-H3K9me3. Importantly, we observed *DNM2*-inhibitors suppresses proliferation of leukemia cells and is synergistic with CK2-inhibitors. Our findings suggest an oncogenic role for high *DNM2* expression in ALL, a possible therapeutic role for *DNM2*–inhibitors alone or combined with CK2 inhibitors in B-cell ALL (Fig. 8).

Others have reported *DNM2* mutations in adults with ALL but *DNM2* expression was not studied^5^,^6^,^26^. Although we did not detect *DNM2* mutations in adults with B-cell ALL (data not shown) we found significantly higher expression of *DNM2*. In contrast, we detected *DNM2* mutations in 4 adults with T-cell ALL^27^. High *DNM2* expression is also seen in subjects with T-cell ALL, but *DNM2* expression in subjects with mutations is difficult to evaluate because of few subjects. Although we found clinical correlations between DNM2 expression and laboratory variables in B-cell ALL we did not find this in T-cell ALL, perhaps because the few subjects studied gave us little power to detect correlations.

High *DNM2* expression is reported in human cancers and is associated with cancer progression and metastasis^28^,^29^, and cancer cell proliferation^30^. Oncogenic mechanisms underlying high *DNM2* expression are partially understood. For example, DNM2 enzymatic activity and proper intra-cellular localization are required for extracellular matrix degradation by invasive cancer cells^31^. *DNM2* is required for the endocytosis of several proteins associated with cancer motility and invasiveness^32^,^33^ and for endocytosis of several oncogenic receptors^34^,^35^. Furthermore, *DNM2* regulates Golgi structure and vesiculation during the secretory process which can affect trafficking of other carcinogenic signaling molecules^36^. Finally, *DNM2* interacts with F-actin and actin dynamics, actin-associated proteins, and molecules that induce or sense membrane curvature as well as expression of angiogenic receptors^37^,^38^. Our study shows high *DNM2*-expression in persons with ALL. We and others think *DNM2* may be important to regulate membrane trafficking of oncogenes and/or oncogenic receptors signaling. It is reported that DNM2 is critical for endocytosis and/or internalization of IL7R, T cell receptor (TCR) and Notch1 ligand Delta-like1 (Dll1), which will trigger signaling activation in the receptor-presenting cells and could be important in the development of ALL^4^,^7^,^38^,^39^,^40^,^41^. Moreover, *DNM2* is involved in control of cytokinesis and acts like a signal transducing GTPase affecting transcriptional regulation^42^. We observed the MiTMAB suppresses the cell proliferation of ALL cells, which is consistent with dynamin inhibitor (MiTMAB)-induced apoptosis in human Jurkat T cells^43^. We also observed that *DNM2* is localized predominately in the cytoplasm with a weak nuclear localization in ALL cells (data not shown). These data suggest a possible nuclear mechanism underlying the oncogenic effect of *DNM2* high expression in ALL although it needs to be further clarified.

It is largely unknown how *DMN2* expression is regulated^44^. Our current study suggests that Ikaros suppresses transcription of *DNM2* in leukemia cells. We observed Ikaros directly binds to and suppress *DNM2* expression. Although Ikaros is dysfunctional in ALL, CK2-inhibitor suppresses *DNM2* expression by restoring Ikaros function by chromatin re-modeling. These findings may explain the upstream mechanism underlying high *DNM2* expression. We also observed a synergistic effect of a *DNM2*-inhibitor with a CK2-inhibitor in suppressing proliferation of B-ALL cells. These data suggest a possible role for inhibiting DNM2 as a therapy of adult ALL.

## Materials and Methods

### Subjects and samples

Between June 2008 and June 2015, 123 consecutive subjects with newly-diagnosed ALL (age 14–77 years old) were studied at the First Affiliated Hospital of Nanjing Medical University (72 had B-cell and 51, T-cell ALL). Diagnoses were based on the WHO Diagnosis and Classification of ALL (2008). The written informed consent was provided by all subjects. The study was approved by the Ethics Committee of the First Affiliated Hospital of Nanjing Medical University, Jiangsu Province Hospital, Nanjing, China, with the 1964 Helsinki declaration and its later amendments or comparable ethical standards. There were no studies with animals.

### Therapy

Therapy details are published protocol (CALLG2008)^45^. Induction was with VDCLP (vincristine, daunorubicin, cyclophosphamide, L-asparaginase, prednisone). Early consolidation used CAT (cyclophosphamide, cytarabine, thioguanine), high-dose methotrexate/L-asparaginase, and mitoxantrone. Late consolidation used VDLP (vincristine, daunorubicin, L-asparaginase, prednisone), COATD (cyclophosphamide, vincristine, cytarabine, epipodophyllotoxin and dexamethasone), high-dose methotrexate/L-asparaginase, epipodophyllotoxin andcytarabine. Maintenance therapy used 6-mercaptopurine and methotrexate. Subjects with *BCR/ABL1*-positive ALL received Imatinib from day 15 of introduction therapy.

### Cytogenetic and molecular analyses

Cytogenetics and detection of the most common Ikaros deletion, Ikaros 6 (Ik6) were analyzed as described^17^. qPCR was performed on StepOne Plus Real-time PCR system (Applied Biosystem-Thermofisher, Foster, CA, USA). Gene expression values of genes of interest (GOI) were achieved in each sample by a formula derived from a scatter graph of Ct values from serial dilutions of a template standard as described^17^,^28^. Expression levels of GOIs were normalized to housekeeping genes expressed as gene expression value of GOI/18 s rRNA. Subjects were allocated in a high or low *DNM2* expression cohort (4^th^ quartile *vs*. 1^st^–3^rd^ quartiles) with a cut-off value (1.619) was determined by SPSS 17.0^17^.

qPCR for *DNM2* expression was analyzed as above in Nalm6, CEM cells and primary ALL cells. Results were normalized to those obtained with *18 s rRNA* and presented as fold-induction over vector controls. Primers: *18s rRNA*, Sense:5′-GTAACCCGTTGAACCCCATT-3′, Anti-sense: 5′-CCATCCAATCGGTAGTAGCG-3′; *DNM2* Sense: 5′-TCAGGACCGGGCTTTTCA-3′, Anti-sense: 5′-CGACCTGCTTTTTCACAATGG-3′.

### Cell culture, plasmids and retroviral gene transfer

The Nalm6 cell line is previously described^46^. The CCRF-CEM (CEM), MOLT-4 and U-937 cell lines were obtained from the American Type Culture Collection (ATCC, Manassas, VA). Cells were cultured in RPMI-1640 medium (Cellgro, Tewksbury, MA, USA) supplemented with 10% fetal bovine serum (Hyclone, Logon, Utah, USA). HEK 293T cells were cultured in DMEM (Cellgro) supplemented with 10% fetal calf serum and 1% L-glutamine (Cellgro). Cells were incubated at 37 °C in a humidified atmosphere with 5% CO_2_. Primary human B- and T-cell ALL cells were cultured in RPMI–1640 medium (Cellgro) supplemented with 10% fetal bovine serum (GE-Hyclone, Logon, Utah, USA). 4,5,6,7-Tetrabromobenzotriazole (TBB) and CX-4945 were purchased from Sigma (St. Louis, MO, USA). Cells were cultured with or without TBB and collected for total RNA isolation. Human *IKZF1* retroviral construct and retroviral production was described^15^,^17^,^47^,^48^.

### Luciferase Assay

Promoter of *DNM2* (−1000bp to +200 bp) was cloned into pGL4.15 vector (Promega, Madison, WI, USA). The transient luciferase assay was performed in HEK293T cells using the Promega luciferase assay reagents and measured with a luminometer according to the manufacturer instructions^15^,^17^. The firefly luciferase activities were calculated as fold-change relative to values obtained from pGL4.15 vector only control cells, and expressed as a percent of pcDNA3.1-*Ikaros* transfection-induced luciferase activity *vs.* the pcDNA3.1 vector. All transfection and reporter assays were performed independently in triplicate at least three times.

### Quantitative Chromatin Immune precipitation (qChIP)

qChIP assays were performed by incubating chromatin with antibodies against Ikaros^15^,^16^,^17^, H3K9me3 (Abcam, Cambridge, MA, USA) or normal rabbit IgG (Abcam) as a control^15^,^16^,^17^,^48^. Enrichment of the ChIP sample over input was evaluated by qPCR with ≥ 3 replicates using specific primers in the promoter region of *DNM2*(forward: 5′-ACCGCGGGATGGAAGAG-3′, reverse: 5′-TGAAGGCGTCCTGCAGTTT-3′). Relative concentration of the qPCR product is presented as the fold change of the level of DNA-Ikaros and DNA-H3K9me3 samples compared with controls.

### *IKZF1* shRNA knockdown

Nalm6 and CEM cells were transiently transfected with human *IKZF1* shRNA constructs in the GFP vector (pGFP-v-RS) (OriGene) using the Neon Transfection System (Invitrogen, Carlsbad, CA, USA). We used scrambled 29-mer shRNA cassette in the pGFP-v-RS vector as a control. Knockdown of Ikaros was confirmed by *IKZF1* mRNA level^15^,^17^,^31^. Primers used for qPCR are 5′-GGCGCGGTGCTCCTCCT-3′ (*IKZF1*-F) and 5′-TCCGACACGCCCTACGACA-3′(*IKZF1*-R).

### Western blot

Cell lysates were prepared as described^15^,^47^,^48^. 20 μg protein was boiled in SDS sample buffer for 10 min., samples run on SDS-PAGE and transferred to the membrane which were blocked with 5% non-fat dry milk at 24 °C for 1 h and incubated overnight at 4 °C with primary antibody (anti-DNM2, 1:1000; Abcam) or anti-actin (1;1000, Santa Cruz, Dallas, TX, USA). The membrane was washed and incubated with goat anti-rabbit IgG conjugated to horseradish peroxidase (1:3000) at 24 °C for 2 h. Blots were developed by the enhanced chemi-luminescence (ECLPlus, Amersham, Arlington Heights, IL, USA) according to the manufacturer’s instructions.

### Cell Proliferation Assay

Cell proliferation was assayed as described^15^,^48^. Briefly, the colorimetric assay (WST-1 reagent) from Roche Life Science (Indianapolis, IN, USA) was performed in 96-well white clear bottom plates (Costar, 3603). 10E + 4 cells were seeded per well with no or PBIT treatment at the indicated concentration and cultured for 72 h. WST-1 reagent was added (10 μl/well) for 4 h and absorbance at 440 nm was measured using a Synergy H1 Hybrid Reader (BioTek. Winooski, VT, USA).

### Statistical analyses

Median differences between the cohorts were evaluated using a Mann–Whitney U-test. Frequency differences were analyzed using uni- and multivariate Cox model. Relapse-free survival (RFS), event-free survival (EFS) and overall survival (OS) were estimated by the Kaplan-Meier method and compared by log-rank test. The Starting point for the observation time for EFS and OS were date of diagnosis. Death in induction, resistance, relapse, death in continuous complete remission or new cancer were considered events in EFS calculation. RFS was estimated for subjects achieving complete remission (CR) starting at the time remission was declared. Living subjects were censored for survival at last follow-up. Statistical analyses used SPSS version 17.0. Data were represented as mean value with bars representing the standard error of the mean (SEM). Determinations of statistical significance were performed using a Student *t*-test for comparisons of two groups or using analysis of variance (ANOVA) for comparing multiple groups.

## Additional Information

**How to cite this article**: Ge, Z. *et al*. Targeting High Dynamin-2 (*DNM2*) Expression by Restoring Ikaros Function in Acute Lymphoblastic Leukemia. *Sci. Rep.* **6**, 38004; doi: 10.1038/srep38004 (2016).

**Publisher’s note:** Springer Nature remains neutral with regard to jurisdictional claims in published maps and institutional affiliations.

## Supplementary Material

Supplemental Figure

## Figures and Tables

**Table 1:** Correlation of *DNM2* expression with clinical and laboratory variables in subjects with B-cell ALL.

	*DNM2* high expression (N = 24)	*DNM2* low expression (N = 48)	Univariate analyses (Chi-Square Tests)	Multivariate analyses (Multivariate Cox model)	
			*P*	P	HR (95% CI)
Age (y; median; range)	40 (14–68)	37 (14–77)	0.645	0.543	0.961–1.078
Male (%)	41.7	58.3	0.182	0.202	0.016–2.392
WBC, × 10^9^/L (median; range)	43 (1–488)	30 (1–398)	0.840	0.504	0.993–1.014
WBC ≥ 30 × 10^9^/L (%)	79.2	41.7	0.003	0.037	4.557 (1.092–19.009)
Haemoglobin, g/L (median; range)	84 (44–150)	95 (48–157)	0.728	0.127	0.933–1.009
Platelets × 10E + 9/L (median; range)	37 (8–292)	45 (4–269)	0.853	0.602	0.991–1.016
LDH (U/L; median; range)	735 (214–7142)	596 (125–4129)	0.625	0.555	0.998–1.001
CD13 + (%)	35.0	55.3	0.142	0.161	0.051–1.640
CD33 + (%)	42.1	47.4	0.707	0.643	0.229–10.903
Hepatomegaly (%)	10.0	4.2	0.714	0.995	0.064–15.889
Splenomegaly (%)	45.8	22.9	0.047	0.059	346.876 (0.803–1.498 × 10^5^)
Lymphadenopathy (%)	60.9	22.7	0.002	0.006	7.245 (1.742–30.134)
*IKZF1* deletion (IK6) (%)	54.2	20.8	0.004	0.016	6.151 (1.401–27.000)
*BCR/ABL1* (%)	39.1	44.4	0.675	0.510	0.225–20.109
Complex karyotype (%)	12.5	23.3	0.623	0.253	0.015–3.024

**Table 2:** Correlation of DNM2 expression with clinical and laboratory variables in subjects with T-ALL.

Characteristics	*DNM2* high expression (N = 8)	*DNM2* low expression(N = 43)	Univariate analyses (Chi-Square Tests)	Multivariate analyses (Multivariate Cox model)	
			*P*	*P*	95% CI
Age (years)	32 (20–55)	28 (14–62)	0.736	0.679	0.877–1.090
Male (%)	75.0	74.4	1.000	0.789	0.093–22.805
WBC, × 10^9^/L median (range)	25.8 (8.7–52.7)	47.9 (3.0–546.0)	0.096	0.210	0.913–1.020
WBC ≥ 30 × 10^9^/L (%)	25.0	63.2	0.113	0.064	0.034–1.098
HGB, g/L median (range)	123 (63–153)	114 (56–167)	0.766	0.756	0.964–1.052
PLT, × 109/L median (range)	103 (44–223)	56 (17–267)	0.137	0.923	0.982–1.021
LDH (U/L) median (range)	1067 (262–4905)	861 (131–8601)	0.953	0.713	1.000–1.001
CD13 + (%)	33.3	32.3	1.000	0.746	0.130–17.239
CD33 + (%)	33.3	37.5	1.000	0.690	0.057–6.637
Hepatomegaly (%)	0.0	17.1	0.478	0.999	0.000–
Splenomegaly (%)	50.0	44.2	1.000	0.382	0.340–16.613
Lymphadenopathy (%)	62.5	82.1	0.449	0.928	0.081–10.601
complex karyotype (%)	0.0	11.8	0.726	0.999	0.000–
