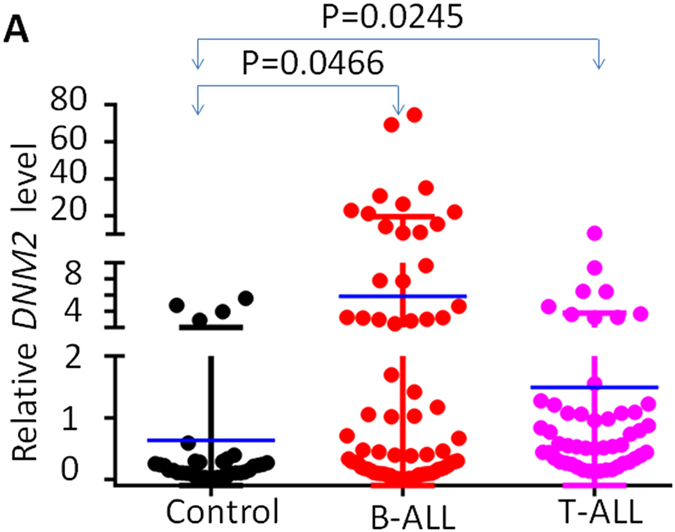# Corrigendum: Targeting High Dynamin-2 (*DNM2*) Expression by Restoring Ikaros Function in Acute Lymphoblastic Leukemia

**DOI:** 10.1038/srep40457

**Published:** 2017-01-11

**Authors:** Zheng Ge, Yan Gu, Qi Han, Gang Zhao, Min Li, Jianyong Li, Baoan Chen, Tianyu Sun, Sinisa Dovat, Robert Peter Gale, Chunhua Song

Scientific Reports 6: Article number: 38004; 10.1038/srep38004 published online: 11 25 2016 updated: 01 11 2017.

This Article contains an error in Figure 1A, where the y-axis ‘Relative DNM2 level’ is incorrectly labelled as ‘Relative CRLF2 level’. The correct Figure 1A appears below as Figure 1.

## Figures and Tables

**Figure 1:**