# Supplementary material for: Targeting High Dynamin-2 (DNM2) Expression by Restoring Ikaros Function in Acute Lymphoblastic Leukemia
Source: Sci Rep. 2016 Nov 25;6:38004. doi: 10.1038/srep38004 (PMC5122860; doi:10.1038/srep38004)
Supplement: Supplemental Figure [file srep38004-s1.pdf]

Targeting High Dynamin-2 (*DNM2*) Expression by Restoring Ikaros Function in Acute Lymphoblastic Leukemia

Zheng Ge[1], Yan Gu[2], Qi Han[2],Gang Zhao[1], Min Li[2], Jianyong Li[2], Baoan Chen[1], Tianyu Sun[4], Sinisa Dovat[3], Robert Peter Gale[5], and Chunhua Song[3]

[1]Department of Hematology, Zhongda Hospital, Medical School of Southeast University, Nanjing 210009, China; [2]Department of Hematology, The First Affiliated Hospital of Nanjing Medical University, Jiangsu Province Hospital, Nanjing 210029, China;
[3]Department of Pediatrics, Pennsylvania State University Medical College, Hershey, PA 17033, USA;
[4]Lewis Katz School of Medicine, Temple University, Philadelphia, PA 19140, USA;
[5]Haematology Research Center, Division of Experimental Medicine, Department of Medicine, Imperial College London, London, SW7 2AZ, UK.

Keywords: *DNM2*; Ikaros; *IKZF1*, adult; acute lymphoblastic leukemia

Running title: *DNM2* and Ikaros in adult ALL

Correspondence
Zheng Ge, M.D., Ph.D.
Zhongda Hospital, Medical School of Southeast University
Department of Hematology
Nanjing 210009, China
Telephone: 86-25-83262468;
FAX: 86-25-83262471
E-mail: Janege879@hotmail.com

Chunhua Song, M.D., Ph.D.
Pennsylvania State University College of Medicine
Department of Pediatrics
500 University Drive, Hershey, PA 17033, USA
Telephone: 717-531-1841;
FAX: 717-531-4789
E-mail:csong@hmc.psu.edu

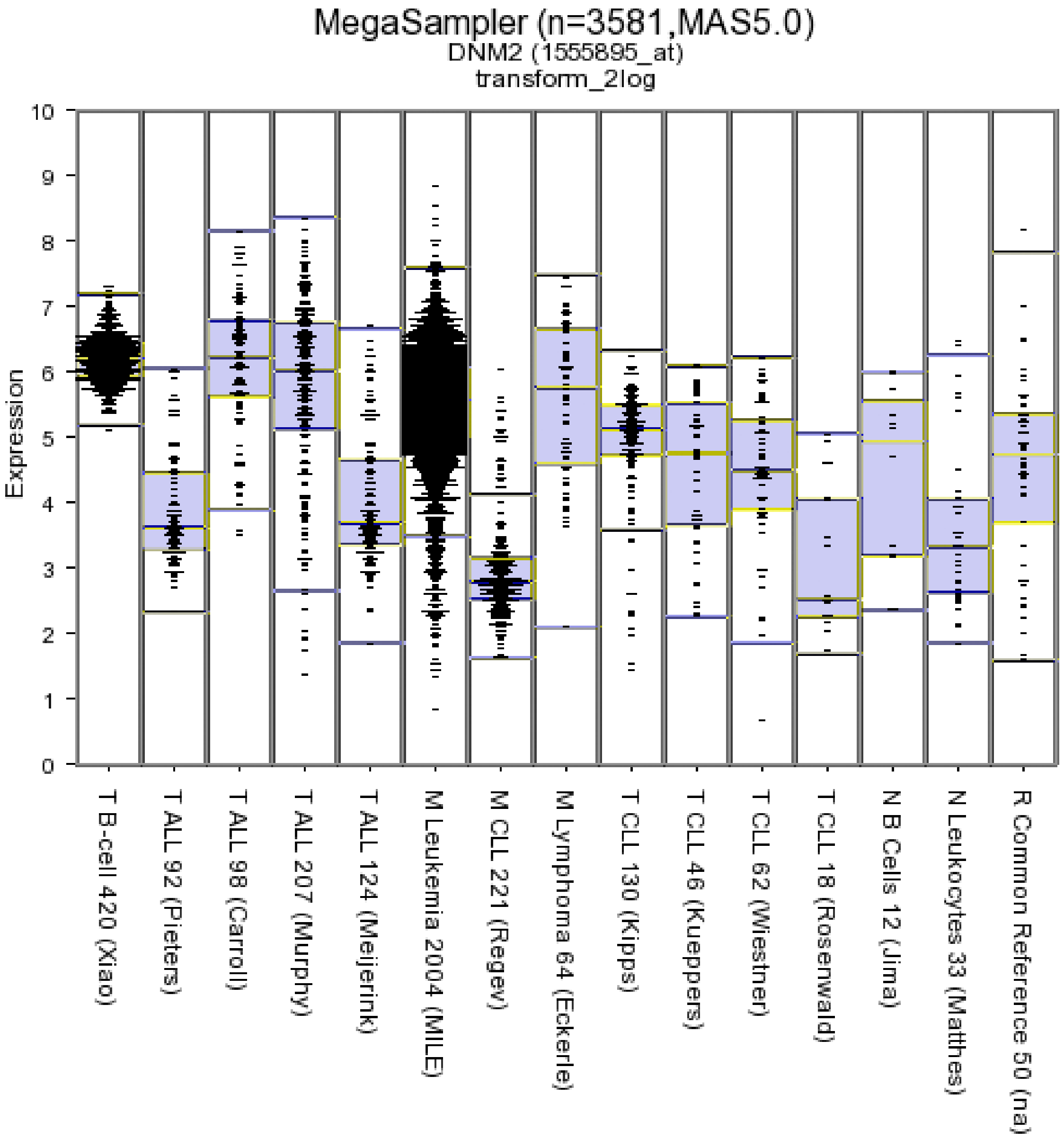

Supplemental Fig 1. DNM2 high expression in ALL cohort study
The microarray data for DNM2 (1555895_at) expression generated with MegaSampler (n=3581, MAS5.0) from human oncogenomics sever crossing all microarray database, which includes B-ALL, T-ALL, Mixed ALL (M ALL), T-CLL, and normal control (N B cells, N leukocytes and common reference) cohort studies.

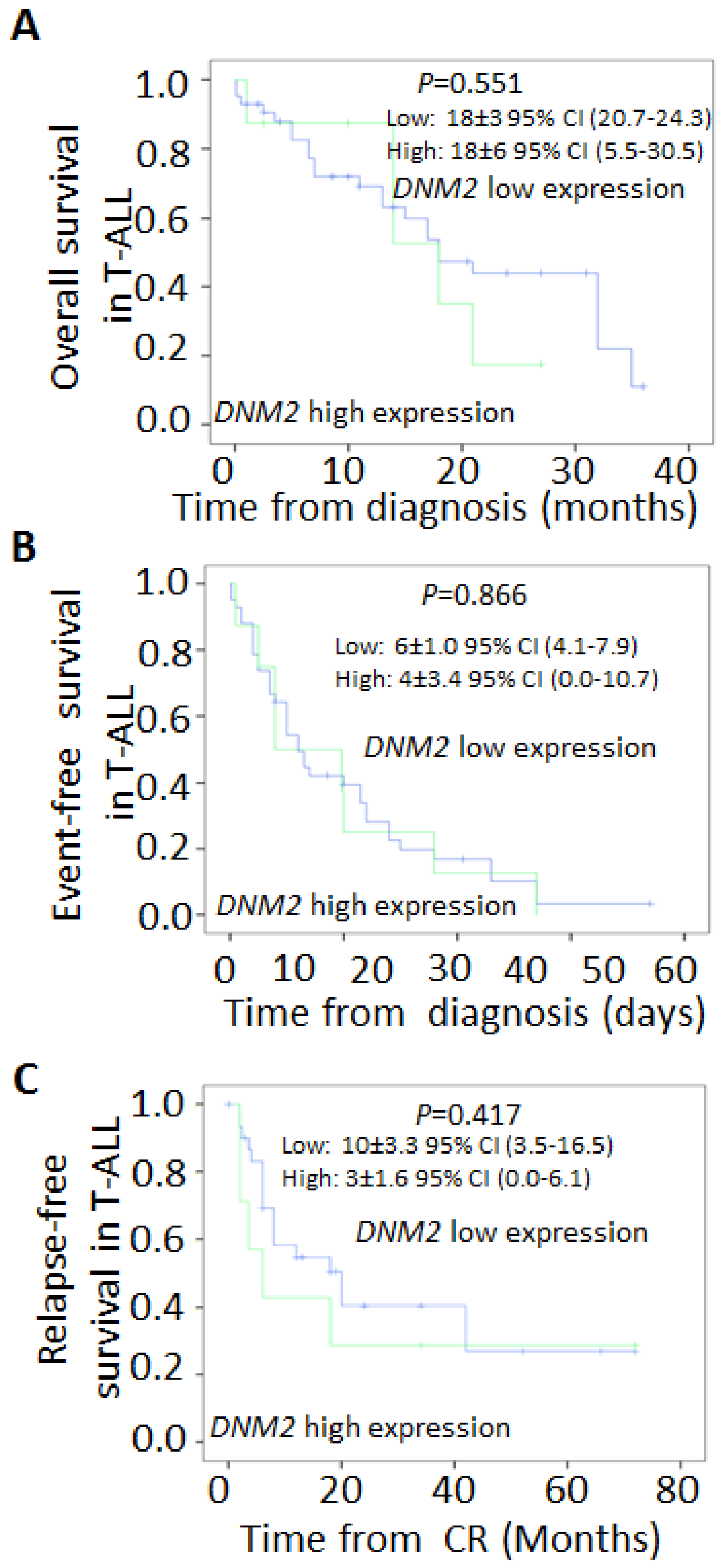

Supplemental Fig 2 Clinical Features of DNM2 high expression in T-ALL

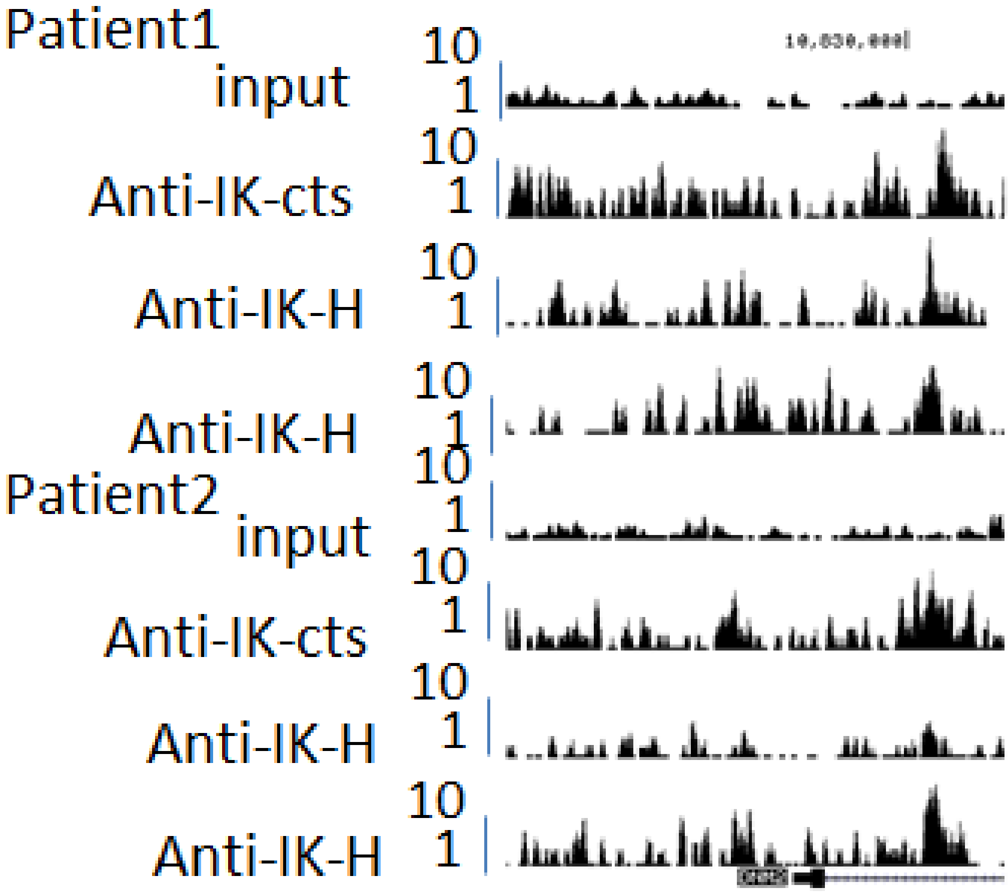

Supplemental Fig 3 Ikaros binds to the promoter of DNM2 in primary B-ALL cells from different patients identified by ChIP-seq (21). Anti-IK-cts: anti-Ikaros c-terminal antibody; anti-IK-H: anti-Ikaros N-terminal antibody .

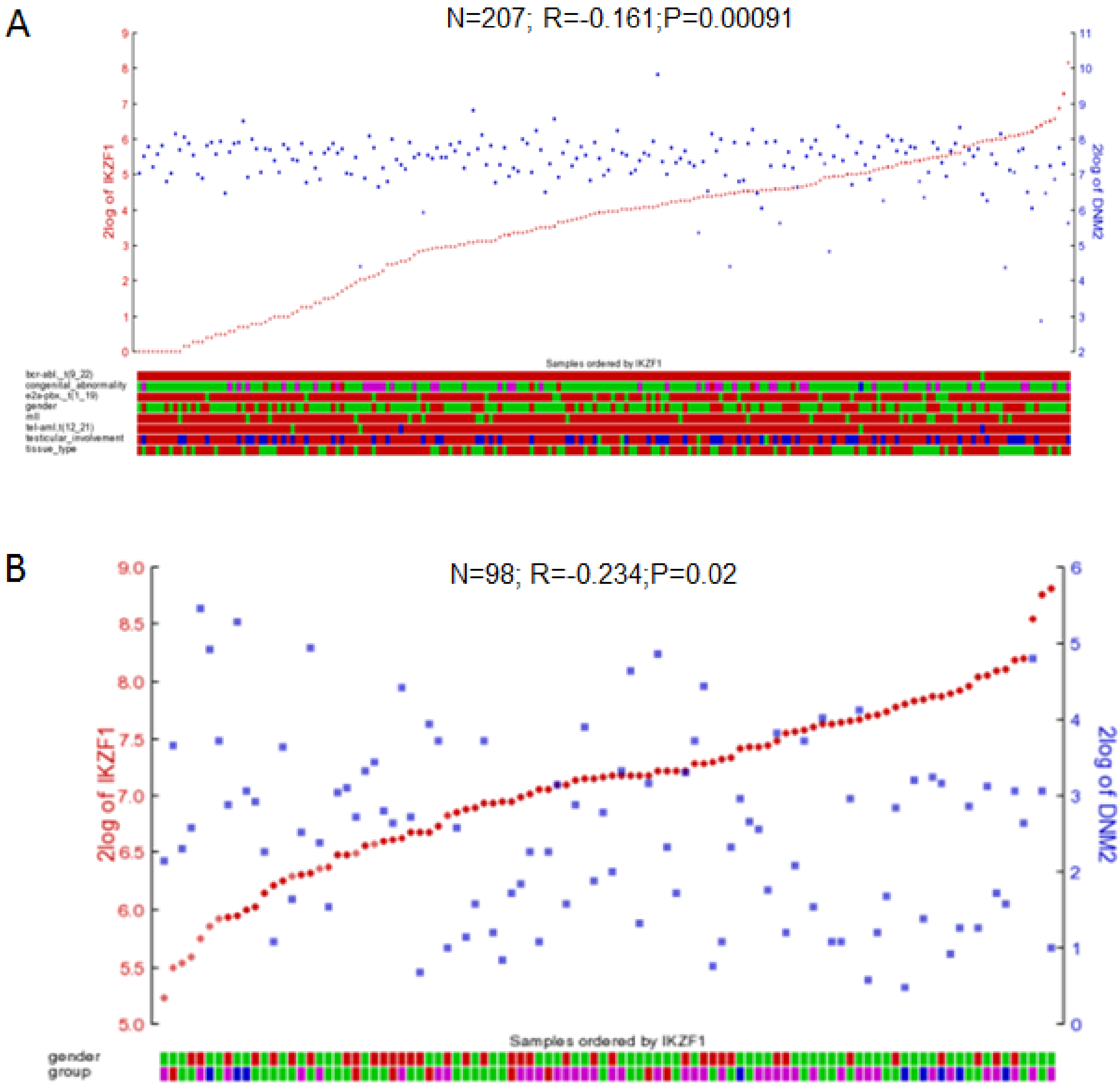

Supplemental Fig 4 Negative correlation of Ikaros expression with DNM2 in 207 (A) and 98 (B) B-ALL patients cohort. Data generated from human oncogenomics sever with GEO database (GSE11877 and GPL15088)

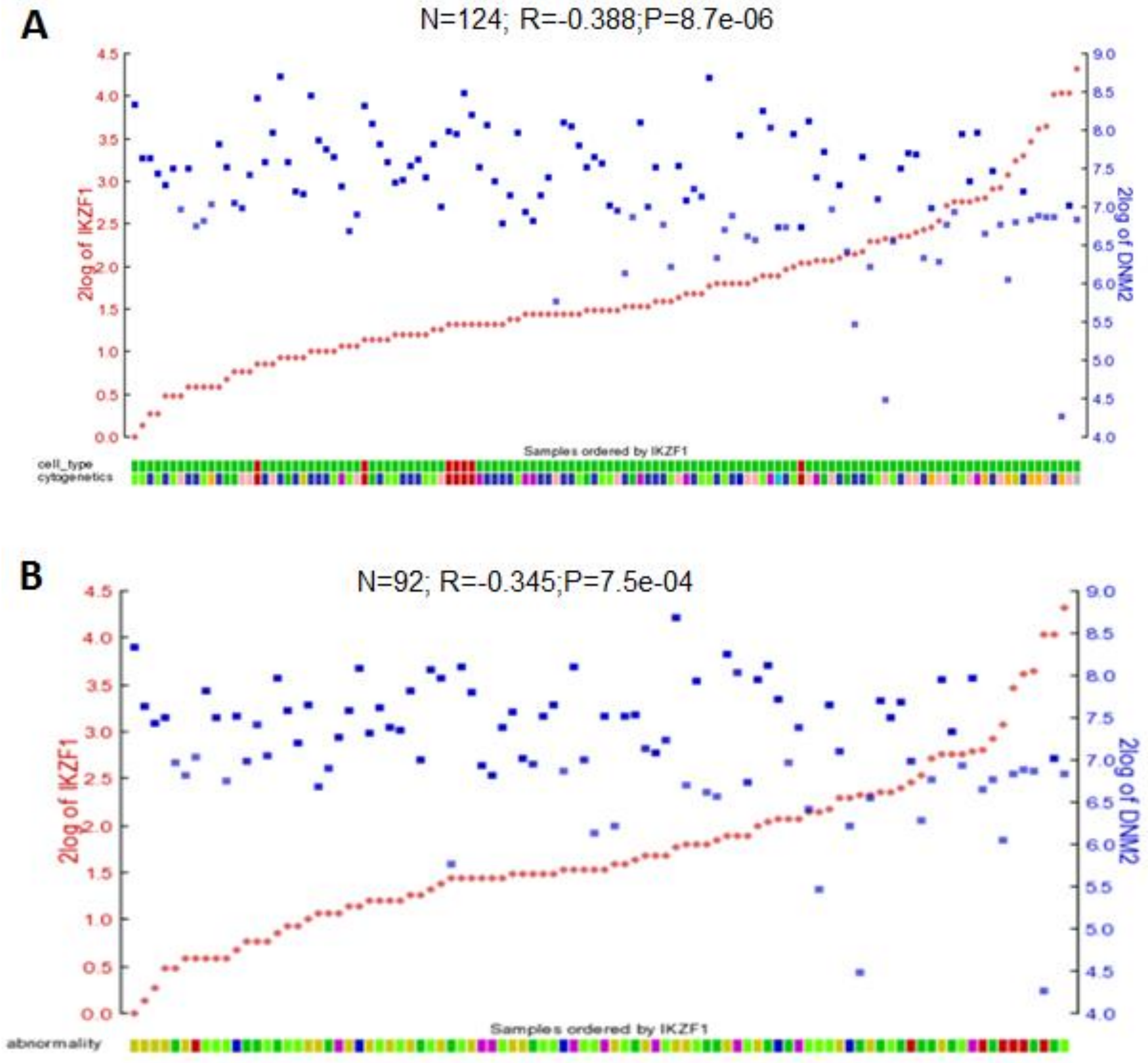

Supplemental Fig 5 Negative correlation of Ikaros expression with DNM2 in 124 (A) and 92 (B) T-ALL patients cohort. Data generated from human oncogenomics sever with GEO database (GPL570 and GSM267399)
